# vFitness: a web-based computing tool for improving estimation of *in vitro *HIV-1 fitness experiments

**DOI:** 10.1186/1471-2105-11-261

**Published:** 2010-05-18

**Authors:** Jingming Ma, Carrie Dykes, Tao Wu, Yangxin Huang, Lisa Demeter, Hulin Wu

**Affiliations:** 1 Department of Biostatistics and Computational Biology, School of Medicine and Dentistry, University of Rochester, Rochester, NY 14642, USA; 2 Department of Medicine, School of Medicine and Dentistry, University of Rochester, Rochester, NY, USA; 3 Department of Epidemiology and Biostatistics, College of Public Health, University of South Florida, Tampa, FL, USA

## Abstract

**Background:**

The replication rate (or fitness) between viral variants has been investigated *in vivo *and *in vitro *for human immunodeficiency virus (HIV). HIV fitness plays an important role in the development and persistence of drug resistance. The accurate estimation of viral fitness relies on complicated computations based on statistical methods. This calls for tools that are easy to access and intuitive to use for various experiments of viral fitness.

**Results:**

Based on a mathematical model and several statistical methods (least-squares approach and measurement error models), a Web-based computing tool has been developed for improving estimation of virus fitness in growth competition assays of human immunodeficiency virus type 1 (HIV-1).

**Conclusions:**

Unlike the two-point calculation used in previous studies, the estimation here uses linear regression methods with all observed data in the competition experiment to more accurately estimate relative viral fitness parameters. The dilution factor is introduced for making the computational tool more flexible to accommodate various experimental conditions. This Web-based tool is implemented in C# language with Microsoft ASP.NET, and is publicly available on the Web at http://bis.urmc.rochester.edu/vFitness/.

## Background

The replication rate (or fitness) between viral variants has been investigated *in vivo *[1,2] and *in vitro *[3-7] for human immunodeficiency virus (HIV). The lack of a consensus on how to measure fitness makes it difficult to determine if the replication capacity is important in disease progression. An accurate method to calculate fitness along with an easy to use tool will be valuable to virologists who study virus fitness.

Although the importance of HIV fitness in disease progression is unknown, the fitness itself plays an important role in drug resistance [8]. In order to develop a better understanding of viral fitness, Marée et al. proposed a mathematical model to describe the dynamics of viral competition between a wild-type virus and a mutant virus, and presented a formula to calculate the relative fitness 1+*s *based on data collected from two time points during the course of the experiment [6]. Here, *s *is the selection coefficient [9]. If there are more than two time points, investigators must choose a pair of time points for the calculation of relative fitness, and the formula does not provide a way to obtain a more accurate estimation over all the observed data. Bonhoeffer et al. proposed a more complicated approach for estimation of viral fitness from time-series data [3] based on the work of Marée et al [6]. Most recently, Wu et al. combined a mathematical model and statistical methods for estimation of virus fitness in growth competition assays [7], which is more in line with population biologist's definition of fitness [9] than the work of Marée et al. [6].

In this paper, we present a Web-based computing tool based on linear regression methods for improving the estimation of *in vitro *HIV-1 virus fitness measured by the growth competition experiment [7]. We will briefly describe the methods and models used in this computing tool, including the growth competition experimental design, a differential equation model, the least-squares regression, and the linear regression with measurement error. Then we will describe software specifications, like the graphic user interface for the estimation, and dilution factors for various experiments. With the data from two experiments of *in vitro *HIV-1 growth competition assay, we use this Web-based tool to estimate the fitness parameters and compare the estimation results with two-point calculations used in previous studies. The Web-based tool is implemented in C# with Microsoft ASP.NET. We also implemented validation controls into the web interface to help users input the correct data. The two-point calculation of virus fitness is also provided in this tool for the purpose of comparison.

## Implementation

### Growth Competition Assay of HIV-1

A growth competition assay developed by Dykes et al. is used here to measure HIV-1 replication fitness by using flow cytometry to determine the relative proportion of test (mutant) and reference (wild-type) viruses [4]. PM1 cells were infected with two virus stocks, each virus expressed a unique marker for expression that is detected on the surface of the infected cell. After 1 hour incubation at 37°C, unbound viruses were washed out with phosphate-buffered saline (PBS). Cells were then seeded in medium and cultured at 37°C. Half of the culture was removed and fresh medium were added in the culture on day 3, 4, 5, and 6. Cells removed from culture were stained with antibodies specific to the markers for infection, and fixed before analysis by flow cytometry. The numbers of wild-type or mutant infected cells are calculated by multiplying the percentage of cells determined by flow cytometry with the absolute number of viable cells in the culture measureed by automated cell counting.

### Modeling

Nowak and May have discussed the general forms of virus dynamics in their book [10], and some simple mathematical models have been used for the estimation of relative fitness for HIV-1 virus fitness experiments [1,3,6]. Wu et al. have used a mathematical model of five ordinary differential equations with five compartments, uninfected target cells (T), cells infected by mutant virus (T_m_), cells infected by wild-type virus (T_w_), number of mutant viruses (M), and number of wide-type viruses (W) [7]. The model can be simplified to three equations involving T, T_m_, and T_w _under quasi steady state (QSS) which assumes that the free virus is proportional to the number of infected cells. Under the assumption of QSS two equations about the change rate of infected cells can be written in the following form [7],(1)

where *δ*_m _represents the death rate of T_m_, and *δ*_w _the death rate of T_w_. If we assume that the number of target cells is constant, integrating Equations (1a) and (1b) over the time period from t_1 _to t_2 _will yield(2)

where Δ_t _= t_2 _- t_1_. By introducing *g*_*m *_= *k*_*m*_*T *- *δ*_*m *_and *g*_*w *_= *k*_*w*_*T *- *δ*_*w *_for the net growth rates of mutant and wild-type infected cells, we have the following three formulas based on two data points to measure fitness parameters,(3)(4)(5)

where *p *is the production rate ratio, *r *the log fitness ratio, and *d *the log relative fitness. And the relative fitness 1*+s *is calculated as(6)

where *s *is the selection coefficient [9].

### Linear Regression

#### Multiple data points

For the growth competition experiments with more than two observations we will use statistical methods to get more accurate estimations of virus fitness. Let *t*_*i *_be the time-point of the *i*^*th *^observation for *T*_m _and *T*_w _(*i *= 0, 1, ..., N-1), and Δ*t*_*j *_be the time interval *t*_*j *_- *t*_0 _(*j *= 1, ..., N-1). We also introduce two variables as follows,(7)

Then, the general form of Equation (3) can be written as(8)

where two variables *m*_*j *_and *w*_*j *_form a linear relationship. Therefore, we know that the parameter *p *can be estimated by linear regression with the observed values of wild-type infected cells and mutant infected cells. Similarly, we can use the linear regression method to get the estimations for parameters *r *and *d*. Finally, the relative fitness 1+*s *can be estimated by exp(*d*) as indicated in Eq.(6). The following sections will briefly list two linear regression methods, the least-squares approach and the measurement error models, which will be used in our computation tool.

#### Least-squares approach

The term linear regression refers to the fact that correlation and regression measure only a linear relationship between two variables. The typical linear regression model without intercept is described as(9)

where *x*_*i *_is the predictor variable, *Y*_*i *_the observed response, and *ε*_*i *_the random error with a normal distribution of *N *(0, *σ*_*ε*_^2^). According to the least-squares approach, the estimation of parameter *β *can be expressed as(10)

#### Linear regression with measurement errors

The measurement error models can be seen in statistical literatures [11,12]. If the measurement errors follow normal distribution and are independent of each other, linear regression with measurement errors can be written as follows [12],(11)

Equation (11-1) is a specification of classical regression, but the true explanatory variable *x*_*i *_is not observed directly. X_i _in Eq.(11-2) denotes the observed measure *x*_*i*_. With the following notations of sample variance and covariance,(12)

the regression coefficient *β *in Eq.(11) can be estimated in two cases: when the ratio of measurement variances is known, or when the measurement variance is known. If the ratio  is known, the estimation of *β *is(13)

If the variance of the measurement error in covariate, , is known, the estimation of *β *is(14)

where(15)

For most biologists who are interested in virus fitness, using those formulas to calculate the regression coefficient would be cumbersome, time-consuming, and impractical. Therefore, we developed a Web-based computing tool, vFitness. Investigators can use different statistical methods to improve the estimation of viral fitness.

### Software Development

#### Web application

We have implemented a Web-based computing tool in C# language with ASP.NET under Microsoft .NET Framework, which provides a means to program Web pages on the Web server facilities of Internet Information Services (IIS). The code of this computing tool runs on the server machine, and investigators can use their web browser to estimate fitness.

#### Graphic user interface

This computing tool provides the graphic user interface for investigators to estimate the relative fitness in competition experiments. Investigators just need to type in the observed values for wild-type infected cells and mutant infected cells in the required format (values delimited by comma), along with the parameters (*δ*_m_, *δ*_w_). Then, the estimation of virus fitness can be easily obtained by submitting the calculation request. This computing tool also provides the validation controls to help users to input correct values for calculation. Four types of validation controls (*Range, Compare, RequiredField, RegularExpression*) have been used to verify the input values. For example, an error message will show up if the observations of *T*_m _are not delimited by commas. The server code also verifies the input values for error checking. One validation is to make sure that the number of time-points is equal to the number of observations.

#### Dilution factor

Since the experimental design involves replacing half the culture with fresh media at each time point, we developed the graphic interface to accommodate the half dilution in growth competition assays and the other dilutions as well.

For an *in vitro *growth competition assay with a half dilution [4,6], half the medium is taken out from the culture for counting and then thrown away at each time point. The observed data are the data from the half volume. So, the total infected cells in the initial culture would be two times the observed data, which results in a dilution factor of 2. The calculation model here is based on the total number of infected cells relative to the initial culture. The only exception is the estimation of parameter d, which depends on the ratio of two observations *T*_m _and *T*_w _at the same time-point in Eq.(5). Two examples of the dilution factor are given as follows,

• If the half dilution is taken at every time point of Day 3, 4, and 5, the corresponding dilution factors would be 2, 4, and 8;

• If one third of testing medium is taken away for counting at each time point of Day 3, 4, 5, and 6, the dilution factors would be **3**, **4.5 **(or 9/2), **6.75 **(or 27/4), and **10.125 **(or 81/8).

#### Missing data

If a dataset is missing at one time point, we can ignore it and continue to estimate fitness parameters with the rest of data. For example, if the data from Day 4 of a 5-day experiment on Days 3, 4, 5, 6, and 7 (half dilution at each time point) was missing, the dilution factors from Day 3 to Day 5 would be 2 to 8 since an additional dilution was made on Day 4.

Note that the above case is different from the case of four observations at Day 3, 5, 6, and 7, in which no dilution takes place on Day 4 and the dilution factors are still 2, 4, 8, and 16.

#### Software deployment

This Web-based computing tool has been deployed on a server computer where the Windows 2003 operating system is running. The web server must run IIS (Internet Information Services), FrontPage Server Extensions and must have the .Net Framework installed. This computing tool can be freely used on the Web at http://bis.urmc.rochester.edu/vFitness/.

## Results

### HIV-1 replication fitness experiments

The growth competition assay mentioned above has been used for the experiments of HIV replication fitness in cell culture [4]. Seven million PM1 cells were infected by a total of 300 ng viruses at a ratio of 75% mutant and 25% wild-type. AT2V106I mutant virus is used in one experiment, and AT2Y188C mutant virus in the other. The same wild-type virus AT1WT is used in both experiments. On day 3, 4, 5, and 6, half of the culture was removed and replaced with fresh medium. Cells removed from culture were measured by a flow cytometer. Table 1 and Table 2 show the measurements for the mutant infected cells *T*_m _and the wild-type infected cells *T*_w _in those two experiments, respectively. The dilution factors (2, 4, 8, 16) have been applied at all time-points to keep the same concentration relative to the initial culture.

**Table 1:** Observation of infected cells in AT1WT/AT2V106I fitness test

Number of infected cells	Day 3	Day 4	Day 5	Day 6
**T**_w_	62,169	161,525	1,293,863	823,368
**T**_m_	284,200	410,025	4,955,738	5,325,275

**Table 2:** Observation of infected cells in AT1WT/AT2Y188C fitness test

Number of infected cells	Day 3	Day 4	Day 5	Day 6
**T**_w_	47,469	140,175	831,250	645,299
**T**_m_	199,369	334,863	1,323,350	1,408,831

### Fitness estimation by statistical methods

Both experiments here have four time points. This computing tool can be easily used for getting the fitness estimation over all observations based on three approaches of linear regression, the least-squares approach (LS), the measurement error model with variance ratio known (MEr), and the measurement error model with variance known (MEv). We set *δ*_m _= 0.5 and *δ*_w _= 0.5 for all estimations (the same death rate chosen in [6], more discussions seen in [13]), *ρ *= 1 for MEr, and  = 0.2 for MEv. Table 3 and Table 4 show the parameter estimation results with the standard deviation (SD) listed in parentheses from those two experiments, respectively. This computing tool also calculated the fitness parameter based on the average method (AM) [3], in which the average value of the production rate ratio p was calculated on the consecutive pair of time points according to Equation 2.4 in the work of Marée et al. [6]. All three statistical approaches gave a very close estimation for the fitness parameter. The simulation analysis in the work of Wu et al. has already shown that the LS, MEr, and MEv approaches yield better estimation than the AM method in terms of mean squared error [7].

**Table 3:** Fitness estimation from AT1WT/AT2V106I experiment

Method	*p *(SD)	*r *(SD)	*d *(SD)	1 + *s*
LS	0.9988 (0.0831)	0.9970 (0.106)	0.1157 (0.353)	1.1226
MEr	1.0023 (0.0835)	1.0026 (0.107)		
MEv	0.9974 (0.0836)	0.9945 (0.107)		
AM	1.1862	1.6546	0.1157	1.1226

**Table 4:** Fitness estimation from AT1WT/AT2Y188C

Method	*p *(SD)	*r *(SD)	*d *(SD)	1 + *s*
LS	0.8532 (0.0471)	0.8104 (0.0557)	-0.2181 (0.271)	0.8040
MEr	0.8543 (0.0471)	0.8119 (0.0558)		
MEv	0.8486 (0.0476)	0.8029 (0.0567)		
AM	0.9838	1.0787	-0.2181	0.8040

### Estimation with missing data

As mentioned earlier, the Web-based tool can be used to deal with virus fitness experiments with missing data by setting the dilution factors accordingly. For examples, we analyzed data from the AT1WT/AT2Y188C experiment. One case with data missing on Day 4, the other with data missing on Day 5, where half of the culture was moved away but could not be counted correctly. The dilution factors were 2, 8, and 16 for the first case, and 2, 4, and 16 for the second one. Table 5 shows the estimation results of parameter p for both cases, respectively. The estimations from those two cases of missing data are very close and are also approximately equal to the values shown in Table 4, except for the average method (AM).

**Table 5:** Parameter *p *estimation with missing data in AT1WT/AT2Y188C

Missing data	LS	MEr	MEv	AM
on Day 4	0.8612	0.8620	0.8551	1.0756
on Day 5	0.8773	0.8784	0.8677	0.8645

### Comparison with two-point calculation

With data from the two experiments, we used this computing tool to easily calculate the fitness parameters on all pairs of time points. Table 6 shows the calculation results of the production rate ratio *p *on any pair of two time points. The results vary depending on the time point chosen. We believe this is due to differences in cultural conditions from day to day. Therefore, estimating fitness based on the linear regression methods will be more accurate because it considers all the observations from the assay.

**Table 6:** Parameter *p *based on two-point calculations

Pair of time-points	Day 3 & 4	Day 3 & 5	Day 3 & 6	Day 4 & 5	Day 4 & 6	Day 5 & 6
AT1WT/AT2V106I	0.7261	0.9673	1.0563	1.1256	1.2329	1.7069
AT1WT/AT2Y188C	0.7521	0.8152	0.8943	0.8635	0.9770	1.3360

## Conclusions

We have developed a Web-based computing tool for improving the estimation of HIV-1 fitness. The tool is based on a mathematical model and linear regression methods which use multiple measurements over time. Two experiments of HIV-1 fitness were completed in this study using growth competition (one with AT2V106I mutant virus, and the other with AT2Y188C mutant virus), and the experimental data has been applied to evaluate the fitness estimation by this Web-based computing tool. The least-squares approach and measurement error models fit the fitness estimation of HIV-1 growth competition, even when data points are missing. It provides an easy way to get a more accurate estimation by using all observations in a fitness experiment.

For comparison, this computing tool also provides the two-point calculation used in the previous studies. Our data has shown that the calculation of the fitness parameter can be very different depending on the pair of time points chosen. Therefore, using all time points to calculate fitness will incorporate the variability from day to day. This computing tool is implemented in C# with Microsoft ASP.NET. The tool provides a graphic user interface and validation controls. Introducing the dilution factor makes it more adaptable to different experimental designs. In this study we competed mutant and wild-type viruses. However, it can be used with any two competing strains of virus by letting W represent one of the strains. This computing tool can be freely used on the Web at http://bis.urmc.rochester.edu/vFitness/.

## Availability and requirement

Project name: vFitness

Project home page: http://bis.urmc.rochester.edu/vFitness/

Operating system: Platform independent, Web application

Program language: C# with ASP.NET

Any restrictions to use by non-academics: license needed

## Authors' contributions

JM developed the software and drafted the manuscript. CD and LD designed and carried out the growth competition assays. TW participated in the software development. JM, YH, and HW performed the statistical analysis. All authors read and approved the final manuscript.
